# Root causes and preventability of unintentionally retained foreign objects after surgery: a national expert survey from Switzerland

**DOI:** 10.1186/s13037-023-00366-9

**Published:** 2023-06-09

**Authors:** David Schwappach, Yvonne Pfeiffer

**Affiliations:** 1 Institute of Social and Preventive Medicine, University of Bern, Mittelstrasse 43, 3012 Bern, Switzerland; 2 Harmfree Healthcare, Wädenswil, Switzerland

**Keywords:** Retained foreign objects, Retained surgical item, Patient safety, Expert survey

## Abstract

**Background:**

Retained foreign objects (RFO) after surgery are rare, serious patient safety events. In international comparisons based on routine data, Switzerland had remarkably high RFO rates. The objectives of this study were to 1) explore national key stakeholders’ views on RFO as a safety problem, its preventability and need for action in Switzerland; and 2) to assess their interpretation of Switzerland’s RFO incidence compared to other countries.

**Methods:**

A semi-structured expert survey was conducted among national key representatives, including clinician experts, patient advocates, health administration representatives and other relevant stakeholders (*n* = 21). Data were coded and analyzed to generate themes related to the study questions following a deductive approach.

**Results:**

Experts in this study unequivocally emphasized the tragedy for individual patients affected by RFOs. Productivity pressure and the strong economization of operating rooms were perceived as detrimental to safety culture, which was seen as essential for RFO prevention, specifically by those working in the OR. RFOs were seen as “maximally minimizable” but not completely preventable. There was strong agreement that within country differences in RFO risk between Swiss hospitals existed. On the systems level and compared to other safety issues, RFO were having less urgency for most experts. The international comparison of RFO incidences raised serious skepticism across all groups of experts. The validity of the data was questioned and the dominant interpretation of Switzerland’s high RFO incidence compared to other countries was a “reporting artifact” based on high coding quality in Swiss hospitals. While most experts thought that the published RFO incidence warrants in-depth analysis of the data, there was little agreement about *who’s* role it was to initiate any further activities.

**Conclusions:**

This investigation offers valuable insights into the perspectives of significant stakeholders concerning RFOs, their root causes, and preventability. The findings demonstrate how international comparative safety data are perceived, interpreted, and utilized by national experts to derive conclusive insights.

## Background

Retained foreign objects (RFO) after surgery are rare, serious patient safety events. The reported annual incidence of RFO ranges approximately between 0.01%—0.02% or 1 per 5′000—10′000 surgeries [1–4]. Near misses in which the lost item is recovered intraoperatively before the patient leaves the operating room are much more frequent [5]. Patients undergoing gastrointestinal, thoracic, and multi-cavity operations seem to be at higher risk for RFO [1]. A meta-analysis based on three early retrospective case–control studies (published between 2003 and 2013) reports that seven risk factors synergistically increase RFO risk: intraoperative blood loss, longer duration of operation, more sub-procedures, lack of or incorrect surgical counts, more than one surgical team and unexpected intraoperative factors [6]. A more recent analysis from Japan revealed that some RFOs were attributable to ignoring count discrepancies during surgery [7].

Most RFOs are detected within few days after the surgery and removed during the index hospitalization but some cases have considerable retention times and remain undetected for years [8]. RFO can have significant physical and psychological consequences for patients and their families. For example, in a large cohort study, Verma et al. found that patients with RFO had significantly increased risks for sepsis, pulmonary infection, wound infection, longer length of stay and higher costs [1]. For surgeons and hospitals, RFOs can have considerable legal, economic and reputational impact [5]. As RFOs are deemed largely preventable, they have been classified as “never events” and require mandatory reporting in some countries. For example, they are listed on the National Quality Forum List of Serious Reportable Events in the US [9]. Among surgical never events, RFO together with wrong site surgery are among the most common events [10, 11]. RFO incidence is also included in the AHRQs “patient safety indicators” [12]. It is the only “never event” with an explicit ICD code. However, the occurrence of RFOs has been questioned as an indicator of safety on the facility level as it is strongly connected to surgical productivity and patient case-mix, and lacks association with other measures of surgical safety or quality [13].

The national RFO rate has been selected as one of the patient safety indicators by the OECD Health Care Quality Indicator (HCQI) project already in 2001. As part of the HCQI project, the OECD publishes international comparative data of RFO rates which are extracted from routine data (https://www.oecd-ilibrary.org/sites/b94b4f09-en/index.html?itemId=/content/component/b94b4f09-en) [14]. Based on this data, Switzerland had remarkably high RFO rates in the past years compared to other countries. In the latest published data the RFO rate was 9.1 per 100′000 hospital discharges in Switzerland (https://www.oecd-ilibrary.org/sites/b94b4f09-en/index.html?itemId=/content/component/b94b4f09-en#figure-d1e8160). Using linked data, the Swiss rate is 4.8-times the New Zealand rate (1.9 per 100′000 hospital discharges), 7-times the rate of the Italy (1.3 per 100′000 hospital discharges), and more than double the OCED13 average (4.0 per 100′000 hospital discharges). Using unlinked data, Switzerland’s rate is highest among OECD20 countries, and substantially higher compared to the USA, UK, and Germany. These high RFO rates in international comparisons could be perceived as an alarming indication for an existing safety problem in the Swiss health care system. In order to address safety concerns at the national level, decision makers and stakeholders must acknowledge and reach a consensus on the significance of addressing the particular issue. However, nothing is known about whether key stakeholders in the Swiss health care system perceive RFO to be a significant problem and how they evaluate and interpret the international comparative data.

The objectives of this study were to 1) explore national key stakeholders’ views on RFO as a safety problem, its preventability and need for action in Switzerland; and 2) to assess their evaluation and interpretation of Switzerland’s results within the comparative international OECD data on RFO. To this end, the study collates perspectives of clinical, safety, policy, and administrative experts for investigating the reasoning of important stakeholders and decision-makers on RFO. In more general terms, we aimed to explore and describe a country’s reflexion on internationally comparative patient safety data in order to better understand how change may develop on a specific safety issue.

## Methods

### Design

**Fig. 1:**
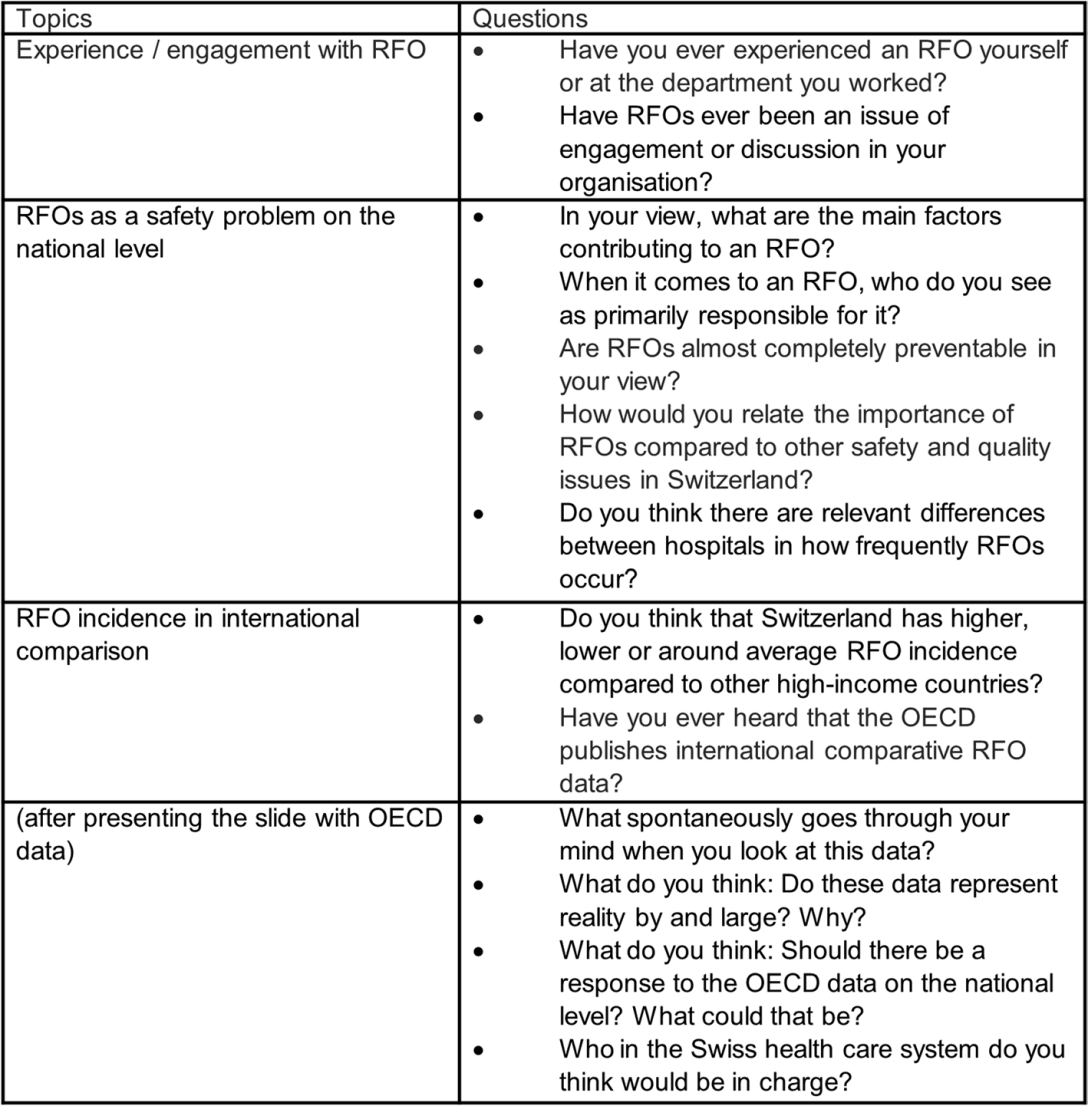
Topic guide for interviews

To explore stakeholders’ perspectives, a semi-structured expert survey was conducted within a person-to-person interview. The survey was developed based on the international literature, national publications and revised based on discussion among researchers. The interview guide closely focussed on answering the study objectives (see Fig. 1).

**Fig. 2:**
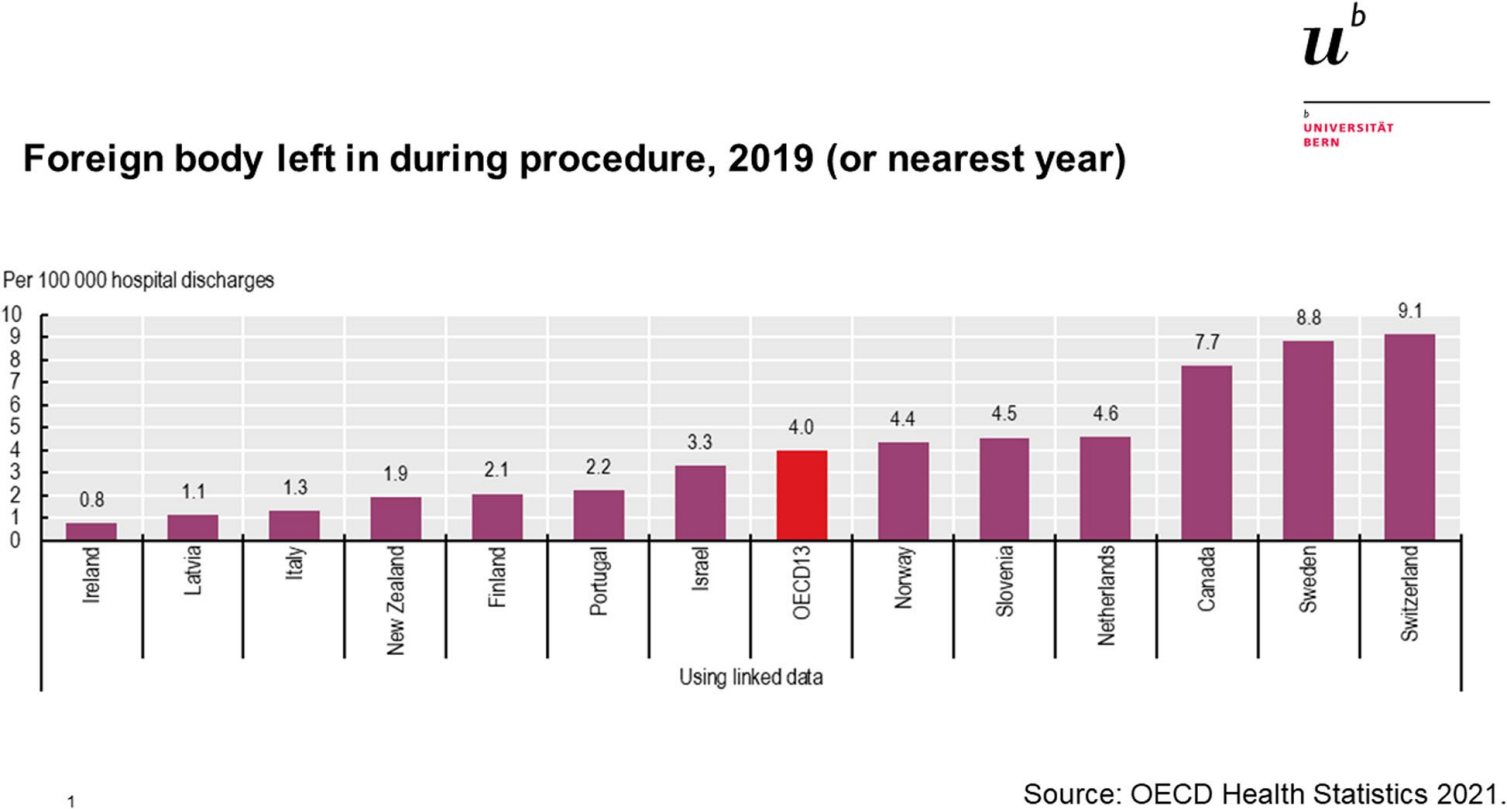
Slide presented to experts during the interviews. The data graph is provided by OECD (https://stat.link/7qtf59)

The interview was structured into two distinct sections: After more general questions related to experts’ views on RFOs, their contributing factors and preventability, experts were presented the original OECD figures on a slide (see Fig. 2) in the second part.

We deliberately showed the OECD data in the second part of the interview, in order to assess the more general views in the first part not influenced by this information. Respondents were informed on the origin of the data, specifically the Swiss data source, and explained the definitions of the indicator, e.g., denominator. They were then asked to express their thoughts about, responses to and interpretation of these data in the second part of the interview. Interviews were conducted via videoconferencing (except two in person) and were recorded with consent of participants. Recordings were transcribed.

### Analysis

Quantitative data were transferred to a spreadsheet. Transcribed qualitative data were coded and analysed to generate themes related to the study questions following a deductive approach. Both researchers are academic patient safety experts and have extensive experience with quantitative and qualitative data analysis. Thematic analysis was performed by the lead author (DS) and subsequently validated by researcher YP. Themes were iteratively refined. Representative quotes were selected.

### Sample

National key representatives were purposively sampled, covering all groups who could be engaged in approaching RFO as a safety issue of national concern. Experts were identified, received a description of the study and its aims, and were invited for participation in the interview. Representatives of the following organizations were approached: perioperative and surgical associations from disciplines at particular risk for RFO (*n* = 14); national quality of care organizations (*n* = 2); national and cantonal health administration (*n* = 4); hospital associations (*n* = 2); large hospitals’ clinical risk managers (*n* = 4) and patients’ advocacy groups (*n* = 2). In total, 28 experts were identified and invited to participate. Experts were instructed that they were not asked for official consolidated statements of their organizations but for their personal views and experiences.

## Results

Twenty-one interviews could be completed (participation rate 75%). Interviews lasted on average 37 min (range: 26–66 min). Of the participating experts, 10 were clinicians and had a leading role in professional perioperative and surgical associations (CLIN-OR). Among these, seven were surgeons or physicians who perform invasive procedures and three were experts in OR management and OR nursing. Three persons were responsible for quality/safety of the cantonal (2) or national (1) health administration (HEALTH-ADMIN). Two experts each were from national health care quality organizations (Q-ORG), national or regional hospital associations (HOSP-ASSO), and patient advocacy organizations (PAT-ADVO) offering support and counseling services to patients. Finally, two experienced clinical risk managers from large hospitals participated (RISK-MGMT). Of all participants, 11 were currently working as health care providers and two had a clinical training but were no longer working in patient care.

### Experts’ experience and previous engagement with RFOs

Nine of the clinically active experts (82%) had experienced RFO either personally, as supervisor or within their unit / department. Beyond their efforts in daily clinical routines, RFOs have not been an explicit and major issue of discussion or activity at most organizations participants represented. Two HEALTH-ADMIN and one Q-ORG representative reported that RFOs were among the patient safety indicators that may be introduced in the future for routine safety monitoring and had been discussed in this context. RFOs are among the perioperative events recorded in the quality registry of one CLIN-OR. A CLIN-OR was leading the development of guidelines and standards for surgical counting procedures. One expert referred to instances in which his HOSP-ASSO provided advice to management and legal handling of RFO events to member hospitals. Many CLIN-OR experts spontaneously mentioned that efforts to prevent RFO have changed dramatically in the last decades with some surgeons showing some concern about the increasing sophistication of counting procedures, their complexity and required time in the OR.

### RFOs as a safety problem: factors contributing to RFOs

Experts mentioned a variety of factors contributing to the occurrence of an RFO. Lack of standardized counting policies and procedures, specifically with intraoperative handovers in the team, human error in counting and false-negative surgical counts were seen as an important risk factors for RFO by CLIN-OR and Q-ORG representatives.

*If you have two hundred plus cloths, and the OR team changes three times, and there is no count at shift changes – you make the door wide open.*

Quote CLIN-OR nursing representative (id 9)

Time pressure, fast-paced work processes and high levels of experienced stress in the OR were one of the most commonly mentioned causes, in particular by CLIN-OR. They were brought forward in the interviews as reasons for loosing items intraoperatively, and for making errors during counting procedures, which itself adds to stress and pressure when it requires time to resolve discrepancies. “Productivity pressure” was clearly very present in the lived experiences of those working in the OR.

*The risk is higher in routine surgeries. It is not the emergencies, it is the routine elective procedures, when you quickly want to get finished. When it goes “fast, fast, fast”, “hurry”. Poorly planned OR times and then there is hurry and stress.*

Quote CLIN-OR OR management representative (id 3)

Culture in the OR, teamworking and communication among staff was another dominant theme that emerged. Experts referred to hierarchy between the different professions and a lack for clarity of roles and responsibilities which could contribute to RFO incidents. Some CLIN-OR experts mentioned past generations of surgeons who would sometimes contribute to tensions between team members in the OR through rude or authoritarian behaviors. Time pressure and culture were sometimes explicitly connected by clinicians bringing up the same example: The surgical count reveals a missing item and a surgeon is hurrying to leave the OR to get to the next surgery. Experts argued that speaking up can be very difficult for OR personnel under such circumstances but would be required to prevent RFOs. Unexperienced or temporal staff and intraoperative changes in the team were seen as factors that further complicated speaking up.

*Information on items may not be handed-over correctly when multiple surgical teams are involved. When a surgeon then tells me "you cannot count" it is difficult to speak up.*

Quote CLIN-OR nursing representative (id 6)

Eighteen experts (including all surgeons) referred to the surgeon as being primarily responsible in case an RFO happens. Three persons each named surgeon and scrub nurse, surgeon and team, or surgeon and hospital.

*As a surgeon, you have to rely on the scrub nurse. If she confirms that everything is clear and the count is correct – what should I do? But I have to go to the patient afterwards to inform him when she lost an item.*

Quote CLIN-OR surgeon representative (id 17)

Three experts (PAT-ADVO; Q-ORG; CLIN-OR nursing) said the entire OR team would be responsible for an RFO without mentioning surgeons explicitly.

### RFOs as a safety problem: preventability of RFOs

All experts agreed that RFOs are largely preventable, but their framing differed. CLIN-OR representatives argued that RFOs could be reduced only to a theoretical minimum while other experts in contrast emphasized their virtually complete preventability.

*Human errors occur – you cannot eliminate it.*

Quote CLIN-OR surgeon representative (id 8)

*Very, very close to 100% preventable.*

Quote HEALTH-ADMIN representative (id 1)

*When you comply with all procedures – it cannot happen.*

Quote RISK-MGMT representative (id 14)

One surgeon representative specialized in high-risk procedures elaborated on deliberate intraoperative decisions to minimize patient harm, which could mean to accept loss of an item temporarily.

*To prevent every single case is difficult. It can be a trade-off. In principle, it is preventable, but you have to consider the consequences. When you have a seriously sick patient in the OR who should go to the ICU immediately, you have to raise the question whether it makes sense for the patient to count cloths for half an hour. But in routine surgeries, there should be no trade-off.*

Quote CLIN-OR surgeon representative (id 4)

A patient advocate made an interesting reference to RFO malpractice cases in which his organization supported patients.

*Patients are not compensated straightforward if it happens. So the legal system obviously does not think that it is completely preventable. But in theory, yes, it is preventable. RFOs could be the type of events that should be directly compensable.*

Quote PAT-ADVO representative (id 7)

RFOs as a safety problem: Relevance of RFO as a safety issue on the national level.

On the systems level and compared to other safety issues, RFO were seen as having less urgency in Switzerland by most experts. The main consideration expressed was that a very small number of patients is affected by RFOs and while for the individual patient the event could be catastrophic and the level of suffering could be substantial, medication safety or surgical site infections put much larger numbers of patients at risk.

*For the specific patient it is a serious problem, but it is not a systematic problem. We are probably at the margin to the maximum achievable safety.*

Quote CLIN-OR physician representative (id 16)

Several CLIN-OR representatives related RFOs and the associated harm to other patient safety issues of more relevance on the systems level, and explicitly named Swiss data protection law and poor health information technology in hospitals. They were arguing that barriers to the fast and easy exchange of relevant clinical information would make it difficult for them to provide safe care, in particular in emergencies and high-risk surgeries.

*There a much more people dying from poor digitization in Switzerland than from RFOs.*

Quote CLIN-OR surgeon representative (id 4)

PAT-ADVO and RISK-MGMT representatives explicitly compared RFOs to diagnostic errors which would be much more frequent but more complex and harder to detect and to approach. One expert of a national quality organization referred to the wider relevance of RFO on the national level.

*Based on the number of events and level of harm it is not so much an important safety issue. But it reflects our culture on how we look at things, and that is why it is important. Because it is so much perceived as an event of individual failure, it is hard to discuss and look at.*

Quote Q-ORG representative (id 2)

All experts, except one PAT-ADVO and a HEALTH-ADMIN representative who both referred to lack of data, unequivocally agreed that they expect relevant differences in the RFO rates between hospitals across the country. Two main arguments were brought forward to substantiate this view: Differences in hospitals’ safety cultures and priorities of local leadership, as well as differing strengths of economic orientation in hospitals.

*What is the main interest of hospitals, economic orientation and production pressure? How far can you squeeze the lemon? Less scrub nurses, less time in the OR. If you have poor work conditions in the OR, more foreign personnel.*

Quote CLIN-OR physician representative (id 16)

*There are cultural differences between hospitals. Hospitals with chief medical officers who have understood the surgical safety checklist make the difference. I expect that more happens at places were the checklist has not been understood.*

Quote RISK-MGMT representative (id 14)

*Absolutely yes; without knowing the numbers; differences in implementation of prevention efforts – resulting from culture.*

Quote HEALTH-ADMIN representative (id 1)

*I expect higher risk in very economically driven institutions; attending surgeons and hospitals may have conflicts of interests. For in-depth analysis of these events I would concentrate on hospitals with a attending surgeon system.*

Quote HOSP-ASSO representative (id 5)

In addition, some CLIN-OR experts mentioned differences in case-mix, general surgical volume, volume of high-risk surgeries, surgical disciplines, surgical team’s experience, and other more clinical aspects that would impact the RFO risk at these specific hospitals.

### RFO incidence in international comparison

All CLIN-OR and HOSP-ASSO representatives believed that Switzerland has lower, or essentially equal RFO incidence rates compared to other high-income countries. Three experts (one HEALTH-ADMIN, one PAT-ADVO and one RISK-MGMT) expected Switzerland to have higher rates. Of the 21 experts, only three were aware that there is international comparative data on RFO incidences available (one HEALTH-ADMIN, one PAT-ADVO and one CLIN-OR). No surgeon was aware of the availability of national data.

When confronted with the slide showing the OECD international ranking by RFO incidence (Fig. 2), most experts were surprised by the Swiss position. Initial spontaneous reactions were dominated by comments on the country’s position relative to others and concerns related to data sources and data quality. This skepticism was prominent across all groups of experts.

*I simply do not believe these figures.*

Quote CLIN-OR surgeon representative (id 4)

*There is no statistical measure of variability or data quality included. So you cannot know whether it is really significant.*

Quote HEALTH-ADMIN representative (id 10)

*What you can see is that Switzerland is very good in reporting [laughter].*

Quote CLIN-OR surgeon representative (id 8)

*Impressive! If that is really the case, we are leaders in the negative sense. Is there a coding effect? What are the others making better? This figure raises several questions.*

Q-ORG representative (id 11)

*Differences in reporting? Are there economic incentives which differ between countries?*

Quote PAT-ADVO representative (id 7)

*This is concerning. Are these cases all clinically relevant to the patient? If these are all patients suffering, that rate is too high.*

CLIN-OR physician representative (id 16)

*The coding quality is very high in Switzerland, in particular if something is reimbursable; If it is not reimbursable in other countries, that could explain the differences.*

Quote HEALTH-ADMIN representative (id 21)

When asked whether the international comparative data by and large reflect reality, two confirmed, fifteen experts clearly declined, and the remaining did not provide an answer. Elaborating on their concerns regarding the validity of the OECD comparison, the main reason expressed was the relation between countries. They believed that Switzerland has high coding standards in hospitals and expected less accurate coding in other countries (i.e., high rates of underreporting). Thus, Switzerland’s position would be an artefact. Some interviewees also mentioned likely underreporting in all countries, including Switzerland, but to varying degrees.

*It is completely biased but we cannot know in which direction.*

Quote CLIN-OR surgeon representative (id 13)

Only two experts (one CLIN-OR surgeon representative and one RISK-MGMT) acknowledged that coding differences were likely to exist but that these would not serve as a sufficient explanation for reported differences between countries.

*We are dramatically worse than 10 other countries. You cannot discuss this away with data quality alone.*

Quote RISK-MGMT representative (id 14)

Experts elaborated to explain differences between countries reported by OECD, based on their personal experience and perception of surgical quality and health care system performance of the comparator countries. This “sensemaking” of the data often focused on Scandinavian countries, the Netherlands, Canada, and Italy (a neighboring country).

*I cannot believe the Italian figures. How is the coding quality? From Netherlands we could learn a lot. The OR staff from the Netherlands is highly educated and oriented towards quality. I can imagine that they have so small numbers, that seems realistic.*

Quote CLIN-OR nursing representative (id 9)

*Switzerland and Netherlands and Canada have probably less underreporting.*

Quote CLIN-OR surgeon representative (id 13)

*Sweden is quite comparable with Switzerland in surgical quality issues. Maybe Canada, Switzerland and Sweden are honest reporters and the others have strong underreporting?*

Quote CLIN-OR surgeon representative (id 17)

*Data from Sweden, the Netherlands and Israel is trustable.*

Quote RISK-MGMT representative (id 18)

*Italy surprises me; the authority of the surgeon in Switzerland is probably higher compared to Sweden, where the culture is more participatory. Could maybe explain the difference?*

Quote PAT-ADVO representative (id 20)

Experts were trying to balance their views on other countries’ levels of surgical safety with their assumptions towards these countries’ coding practices to explain differences in RFO rates. For some countries, low RFO rates were unequivocally explained by poorer reporting, sometimes complemented by reflections on the lower frequency of high-risk surgery performed in these countries. On the other side, the positions of Netherlands, Finland, and Israel, which have considerably lower RFO rate compared to Switzerland on the OECD graph, seemed to be more troubling for CLIN-OR experts. The perceived level of surgical care and coding quality co-existed for these countries. Thus, from these experts perspectives, there was no obvious reason to question the considerably lower RFO rates of these countries. While verbalizing their thoughts, some experts became self-aware that they were trying to selectively explain figures that confirmed their pre-existing views.

### Responding to international RFO data

Independent of the discipline they represented, most experts thought that the publication of the OECD figures should initiate some response on the national level. Deeper investigations and analysis of the data were typically mentioned as a potential first step.

*We have to act upon this. I thought that we are at an incidence so low that it cannot be reduced further. But these figures show we are not there at all.*

Quote RISK-MGMT representative (id 14)

*It is good that we have figures. It requires more in-depth analysis whether we really have a problem.*

Quote HOSP-ASSO representative (id 12)

*This is not satisfying. We cannot simply leave that statistic without response.*

Quote Q-ORG representative (id 11)

Some participants focused the international comparison that would require further analysis to clarify the seize of the problem in Switzerland. Others were orienting on the Swiss numbers “as they stand” and recommended in-depth national analysis and validation studies.

*If we would understand the differences between and within countries—we would know what to do and could solve the problem.*

Quote CLIN-OR surgeon representative (id 4)

*We should investigate the validity of the data: identify clusters, types of surgeries, differences between hospitals and regions, public and private hospitals. And whether there is underreporting; there needs to be a response.*

Quote CLIN-OR surgeon representative (id 17)

Two CLIN-OR experts stated they were unimpressed by the OECD numbers and would not recommend further activities or inquiries because there were other issues of higher priority.

*Everyone makes efforts to reduce it and you will never reduce it to zero. I would not invest too much time and resources on the topic.*

Quote CLIN-OR surgeon representative (id 8)

There was little clarity and no agreement between experts what they expected from different stakeholders in the system and *who* should or could initiate further activities. In particular, there were different views on regulative interventions. While some experts expected regulative bodies on the cantonal level to initiate discussion with hospitals and surgeons,, others were clearly opposed to this approach.

*It is the cantonal regulating bodies’ role to ensure that patients have no elevated risk for RFO regardless of which hospital they go to. The regulative body should take action immediately.*

Quote HEALTH-ADMIN representative (id 1)

*National standards for counting procedures would help to decrease variation; it is a professional issue, not so much a regulative issue.*

Quote CLIN-OR nursing representative (id 9)

*Not efficient if the regulator would engage in that. By law the cantons are responsible, but the cantons are not equipped to do that.*

Quote RISK-MGMT representative (id 14)

Similarly, there was no consensus on the role of professional associations (e.g., surgical associations). Experts questioned the potential power of surgical associations and also argued that there are too many, usually small, organizations involved in Switzerland.

*It does not help much if the professional organizations engage in quality recommendations and guidelines, if nobody controls it. It will only work with economic incentives or control mechanisms.*

Quote CLIN-OR surgeon representative (id 17)

*Professional organizations can only make recommendations. And that is probably not enough?*

Quote CLIN-OR physician representative (id 19)

Hospitals were seen as responsible for efforts to prevent RFO, but whenever they were mentioned, it was acknowledged that external pressure or incentives would be required to trigger specific engagement or improvement activities.

*Hospitals should engage, but the economic incentive runs against more prevention. Maybe explicitly pay for the surgical count, so that you get paid for the time? But recommendations and voluntary actions are not enough.*

Quote PAT-ADVO representative (id 20)

*It requires hospitals and their leadership. But they have so many issues to deal with. A just culture needs to be pushed in hospitals. This would be most sustainable; It will trickle down to the OR teams. But that needs external pressure by cantons and health insurers.*

Quote HEALTH-ADMIN representative (id 21)

Two experts mentioned the federal quality commission (introduced in 2022) and suggested that they could be in the position to advance the topic (both HEALTH-ADMIN). Another CLIN-OR surgeon representative argued that he would like to see researchers and analysts in the field to further explore the data. Overall, experts seemed to have no clear vision and expectation of whose responsibility and role it would be to act based on the international data, if at all, and there was obviously no distinct strategy that appeared potentially successful to them.

## Discussion

This study provides insights into the perspectives of a diverse panel of national experts in the fields of surgical care and patient safety, shedding light on their viewpoints regarding RFO.

Experts in this study unequivocally emphasized the tragedy for affected individual patients. RFOs were seen as “maximally minimizable” but not completely preventable. Some respondents expressed views that RFOs needed to be prevented further and that this issue warranted investment in safety practices while others thought that other safety concerns should be prioritized instead. Productivity pressure and the strong economization of operating rooms were perceived as detrimental to safety culture, which was seen as essential for RFO prevention, specifically by those working in the OR. Consequently, experts hypothesized that hospitals experiencing higher economic pressure may have higher RFO rates. There was strong agreement that within country differences in RFO risk between Swiss hospitals existed. However, RFOs were currently not perceived as a patient safety priority on the national level and most experts believed that Switzerland would have at least average performance in RFO rates compared to other countries.

The international data were largely unknown to the experts and it is surprising that the regular publication of comparative RFO incidences with Switzerland taking a concerning position went unrecognized despite some uptake by the public media. Given the impressive nature of RFO events for the public we expected that experts may have been confronted with these figures previously, which was not case. Irrespective of their role in the health care system (e.g., care provider versus regulator), experts’ dominant interpretation of Switzerland’s high RFO incidence compared to other countries was a “reporting artifact”. The OECD emphasizes that higher adverse event rates may “signal more developed patient safety monitoring systems and a stronger patient safety culture rather than worse care” [14]. However, differences in RFO coding in routine hospital data can only be partly attributable to safety culture. As RFO incidence data is based on routine hospital data coded for billing purposes, it is probably more affected by economic incentives and disincentives to code these events. These effects synergistically make interpretation of the data difficult and systematic differences between countries very likely. This raises doubts about the usefulness of publishing international routine RFO. In contrast, looking back to the development of the preliminary set of quality indicators for international comparisons in 2013, the indicator “retained surgical item or unretrieved device fragment (adult)” obtained the highest rating of being “internationally feasible” among all evaluated indicators and received the maximum achievable rating for recommendation to keep as quality indicator [15] in a Delphi consensus approach of the OECD health care quality indicators expert group.

While the relative position of Switzerland compared to others was regarded as unreasonable, and as not reflecting the size of the problem in comparison between the countries, most experts argued that the national data would warrant further in-depth analysis. The interviewed experts were particularly interested in the surgical procedures involved, the clinical characteristics of the cases and potential differences between types of hospitals. Analysis of the events reported to voluntary or mandatory sentinel reporting systems can provide valuable insights into the multitude of factors contributing to RFO incidents [16]. As reported by clinical risk managers from Swiss hospitals, not all serious events are currently analysed for their underlying causes [17]. Investigation of single RFO events was not suggested as a national strategy to learn from these serious incidents. Research suggests, however, that such in-depth analysis of RFO events can be beneficial to gain a deeper understanding and implementation of procedures based on retention risk classification of surgical items can be successful to prevent RFOs [18].

A strength of this study is the diversity of the expert panel and the high participation rate which indicates interest in the study objective and trust in the researchers. All expert panel members were deliberately chosen based on their extensive experience, comprehensive overview, and presumed independence, with the intention to encourage candid and open expression of opinions. For example, all providers also had a leading role in professional associations. It is evident that clinicians at earlier stages of their career are likely to hold distinct perspectives and experiences, for example, related to culture in the OR and its evolution over the past decades. Given our aim to capture spontaneous and unfiltered responses, we intentionally presented the RFO data during the interview without providing them beforehand for preparation. Reflections that may arise from deeper or prolonged deliberation might not be effectively captured. Finally, we designed the study as individual interviews to ensure that diverse perspectives were fully expressed. While focus groups could have been an alternative approach, as they allow for exchange and discussions among experts, they may carry the risk of minority views or personal experiences not being transparently expressed, particularly in sensitive issues.

Despite these limitations, this research contributes to understanding diverse key stakeholders’ views on RFOs, their causes and preventability. To the authors’ knowledge, this is the first investigation into how international comparative safety data are perceived and interpreted and how important stakeholders reason about them and draw conclusions for actions. Particularly, the multitude of ideas and perspectives on responsibilities and potential modes of action on regulatory and national level illustrates the challenges in driving change in a setting that is under considerable financial stress like surgery in healthcare.

## Conclusions

This investigation shows that the same safety issue and statistics can elicit diverse sensemaking among respondents. Some participants believed that there were other more pressing issues, while others recognized the need for action to further reduce RFOs. Furthermore, certain respondents attributed the RFO occurrence to human error that can never be eradicated, while others viewed it as completely preventable. These variations in sensemaking can have important consequences for the perceived need for action. The results also illustrate that data really need to be compelling to motivate stakeholders to act, as the proposition to look more deeply in the validity of the reported figures was one of the most broadly shared conclusions. Studying sensemaking around important safety issues may be a fruitful avenue for future research in order to identify potential factors conducive to or detrimental for the development of large-scale efforts in reducing patient safety risks.

## Data Availability

The data that support the findings of this study are available on reasonable request from the corresponding author. The data are not publicly available due to privacy and ethical restrictions.
